# Calpain-mediated protein targets in cardiac mitochondria following ischemia–reperfusion

**DOI:** 10.1038/s41598-021-03947-9

**Published:** 2022-01-07

**Authors:** Ling Li, Jeremy Thompson, Ying Hu, Edward J. Lesnefsky, Belinda Willard, Qun Chen

**Affiliations:** 1 Proteomics Core, Cleveland Clinic, Cleveland, OH 44195 USA; 2 Division of Cardiology, Department of Internal Medicine, Virginia Commonwealth University, Richmond, VA 23298 USA; 3 Department of Biochemistry and Molecular Biology, Virginia Commonwealth University, Richmond, VA 23298 USA; 4 Department of Physiology and Biophysics, Virginia Commonwealth University, Richmond, VA 23298 USA; 5 McGuire Department of Veterans Affairs Medical Center, Richmond, VA 23249 USA

**Keywords:** Cell biology, Physiology

## Abstract

Calpain 1 and 2 (CPN1/2) are calcium-dependent cysteine proteases that exist in cytosol and mitochondria. Pharmacologic inhibition of CPN1/2 decreases cardiac injury during ischemia (ISC)–reperfusion (REP) by improving mitochondrial function. However, the protein targets of CPN1/2 activation during ISC–REP are unclear. CPN1/2 include a large subunit and a small regulatory subunit 1 (CPNS1). Genetic deletion of CPNS1 eliminates the activities of both CPN1 and CPN2. Conditional cardiomyocyte specific CPNS1 deletion mice were used in the present study to clarify the role of CPN1/2 activation in mitochondrial damage during ISC–REP with an emphasis on identifying the potential protein targets of CPN1/2. Isolated hearts from wild type (WT) or CPNS1 deletion mice underwent 25 min in vitro global ISC and 30 min REP. Deletion of CPNS1 led to decreased cytosolic and mitochondrial calpain 1 activation compared to WT. Cardiac injury was decreased in CPNS1 deletion mice following ISC–REP as shown by the decreased infarct size compared to WT. Compared to WT, mitochondrial function was improved in CPNS1 deletion mice following ischemia–reperfusion as shown by the improved oxidative phosphorylation and decreased susceptibility to mitochondrial permeability transition pore opening. H_2_O_2_ generation was also decreased in mitochondria from deletion mice following ISC–REP compared to WT. Deletion of CPNS1 also resulted in less cytochrome *c* and truncated apoptosis inducing factor (tAIF) release from mitochondria. Proteomic analysis of the isolated mitochondria showed that deletion of CPNS1 increased the content of proteins functioning in regulation of mitochondrial calcium homeostasis (paraplegin and sarcalumenin) and complex III activity. These results suggest that activation of CPN1 increases cardiac injury during ischemia–reperfusion by impairing mitochondrial function and triggering cytochrome *c* and tAIF release from mitochondria into cytosol.

## Introduction

Calpain 1 (CPN1) and calpain 2 (CPN2) are ubiquitous calpains that exist in cytosol and mitochondria. Activation of CPN1 and CPN2 contributes to cardiac injury during ischemia and reperfusion by cleaving cytosolic and mitochondrial proteins^1–5^. Ischemia–reperfusion leads to mitochondrial dysfunction that becomes a key source of myocyte injury by decreasing ATP production, increasing the generation of reactive oxygen species (ROS), and releasing pro-apoptotic proteins from mitochondria including cytochrome *c* and apoptosis inducing factor (AIF)^2,3,6,7^. Thus, either the attenuation of damage to mitochondria or the timely removal of dysfunctional mitochondria through mitophagy are needed to decrease cardiac injury during ischemia–reperfusion^8–11^. Unfortunately, mitophagy is inhibited during ischemia–reperfusion due to the activation of CPN1^3,12^.

Ischemia–reperfusion impairs the mitochondrial electron transport chain (ETC) and decreases the activities of metabolic enzymes within the mitochondrial matrix^13–15^. Inhibition of CPN1 and 2 protects the ETC^3,16^ and metabolic enzymes including pyruvate dehydrogenase^15^ during ischemia–reperfusion, supporting that activation of mitochondrial calpains contributes to mitochondrial damage. Activation of mitochondrial CPN1 (mCPN1) contributes to the release of AIF from mitochondria into the cytosol which triggers caspase-independent apoptosis during ischemia-reperfusion^2,17,18^. Activation of mitochondrial CPN2 (mCPN2) contributes to complex I damage during ischemia–reperfusion through degradation of the ND6 subunit^16^.

The mitochondrial permeability transition pore (MPTP) is a nonselective pore formed in the inner mitochondrial membrane^19,20^. Although the exact structure of the MPTP remains uncertain, an increased susceptibility to MPTP opening undoubtedly increases cardiac injury during ischemia–reperfusion^19,21^. The opening of MPTP increases cell injury by uncoupling mitochondrial respiration and increasing the permeability of the inner and outer mitochondrial membranes^19^. An accumulation of calcium within mitochondria is a key factor leading to MPTP opening^19^. Calcium overload is also a key factor to activate the CPN1 and 2^17,22^. Activation of the mitochondria-localized CPN1^3,15^ and CPN2^16^, in turn, also favors MPTP opening during ischemia–reperfusion, potentially creating a mutually reinforcing positive feedback loop of calcium mediated mitochondrial damage. Interestingly, most of these conclusions are based on the use of pharmacologic approaches^3,15,16^.

In addition to ischemia–reperfusion, activation of mCPN1 also contributes to complex I damage during endoplasmic reticulum stress^23^. Activation of mCPN1 and mCPN2 are involved in mitochondrial damage in other stress conditions including diabetic cardiomyopathy^24^, doxorubicin-induced cardiotoxicity^25^, and heart failure^1,26^. These results indicate that activation of mitochondrial calpains plays a key role in mitochondrial damage under pathological conditions. Thus, a proteomic approach was used to identify potential targets of mitochondrial calpain activation during ischemia–reperfusion. Ischemia–reperfusion was used as the working model in that this model leads to mitochondrial calcium overload and mitochondrial damage^6^. A genetic approach was used to eliminate the activities of the CPN1 and 2^27–29^ CPN1 and CPN2 contain one large subunit (78 KD) unique to each isoform and one small regulatory subunit (CPNS1) shared by both isoforms^27^. Genetic deletion of CPNS1 eliminates the activities of both CPN1 and CPN2^27^. Thus, cardiomyocyte specific CPNS1 deletion mice with a conditional onset of CPNS1 deletion were used in the present study. A proteomic approach was employed to identify the altered mitochondrial proteins in both wild type (WT) and deletion mice in control and ischemia–reperfusion conditions.

## Results

### Cardiac myocyte specific conditional CPNS1 deletion decreased cytosolic and mitochondrial CPN1/2 activation during ischemia–reperfusion

**Figure 1:**
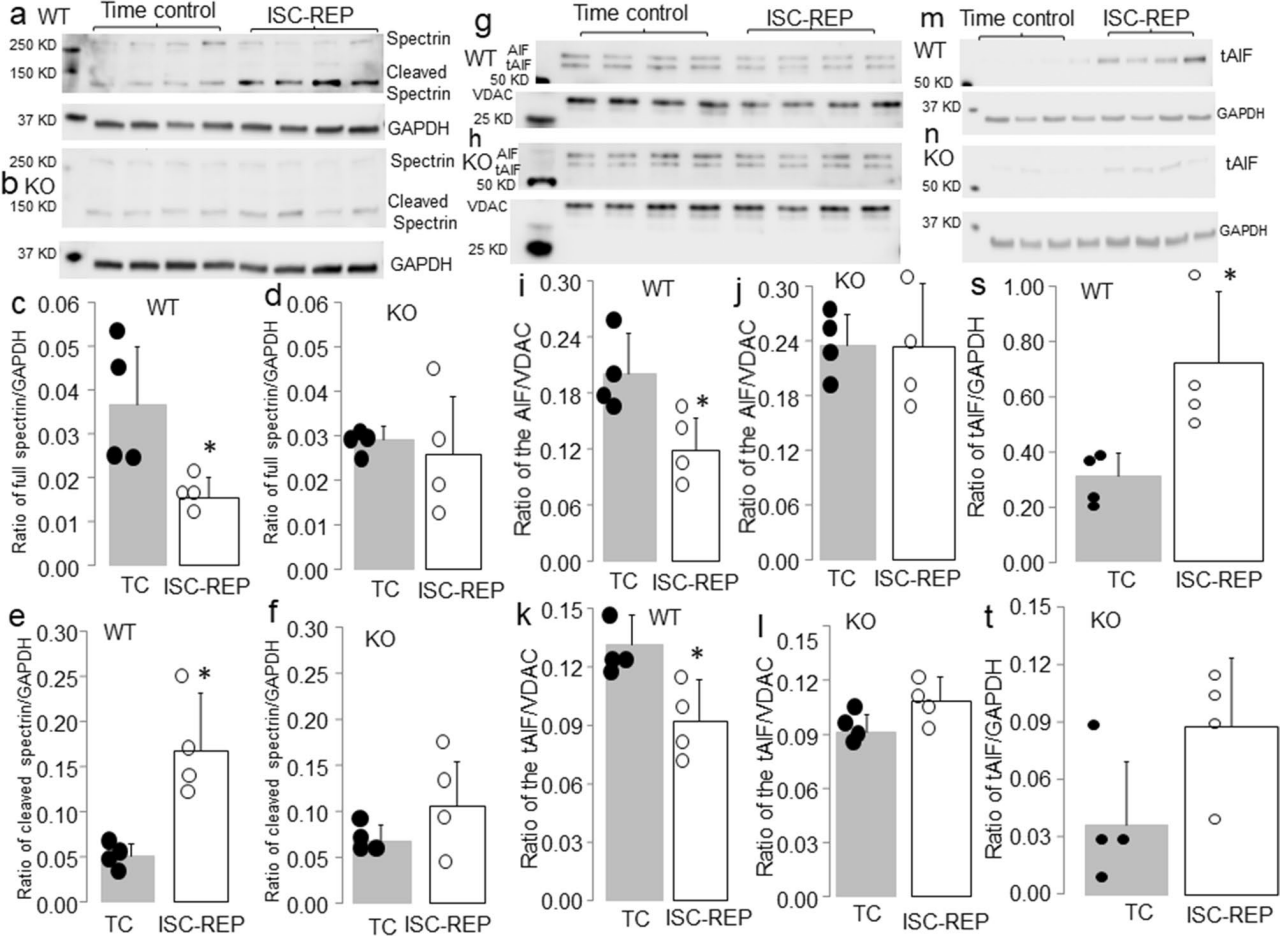
Deletion of CPNS1 prevents cytosolic and mitochondrial CPN1 activation during ischemia (ISC)–reperfusion (REP). In wild type mice, ISC–REP led to decreased spectrin content in cytosol compared to time control (**a**,**c**). The ISC–REP also increased the content of cleaved spectrin in wild type compared to time control (**a**,**e**). These results supported that ISC–REP activated cytosolic CPN1 and CPN2. In contrast, ISC–REP did not alter either spectrin (**b**,**d**) or cleaved spectrin content (**b**,**f**) in CPNS1 deletion mice compared to time control, indicating that elimination of CPNS1 prevented activation of cytosolic CPN1 and CPN2 in deletion mice. In wild type mice, ISC–REP led to decreased contents of AIF (67 KD and 62 KD) compared to time control (**g**,**i**,**k**), supporting that ISC–REP activated mitochondrial CPN1. However, ISC–REP did not alter AIF content (**h**,**j**,**l**) in CPNS1 deletion mice compared to time control, indicating that elimination of CPN4 prevented activation of mitochondrial CPN1 in deletion mice. In wild type mice, ISC–REP led to increased tAIF content in cytosol compared to time control (**m**,**s**). However, ISC–REP did not markedly increase tAIF content in cytosol in deletion mice compared to time control (**n**,**t**). These results suggest that elimination of CPNS1 decreases AIF release from mitochondria into cytosol during ISC–REP. GAPDH was used as protein loading control. Data are expressed as mean ± SD; *p < 0.05 vs. time control. n = 4 in each group. Not every sample was used for immunoblotting in each group.

An increase in the content of cleaved spectrin is used as an indicator of cytosolic CPN1/2 activation^3–5,30^. Ischemia–reperfusion decreased the content of full length spectrin and increased the content of cleaved spectrin in WT mice compared to time control (Fig. 1a,c,e). In contrast, the contents of full length and cleaved spectrin were not altered in deletion mice following ischemia–reperfusion (Fig. 1b,d,f). These results support that elimination of CPNS1 prevents cytosolic CPN1/2 activation during ischemia–reperfusion. As an indicator of the functional activation of mCPN1, we measured AIF and its cleaved form truncated AIF (tAIF) to reflect mitochondrial CPN1 activation^2,17^. AIF is imported into the mitochondrial matrix as a 67 KD precursor and mature AIF (AIF, 62 KD) is formed following removal of the mitochondrial leader sequence^31^. The AIF is bound on the outer leaflet of the inner mitochondrial membrane^17,31–33^. Activation of CPN1 within the mitochondrial intermembrane space cleaves the AIF to tAIF (57KD) that can be released from mitochondria into cytosol when the permeability of outer mitochondrial membrane is increased^15,17,32^. Therefore, a decrease in mature AIF content in mitochondria or an increase in truncated AIF content in the cytosol is used to represent mitochondrial CPN1 activation^2,3,17^. Ischemia–reperfusion decreased the AIF content in WT mice compared to time control (Fig. 1g,i). A decrease in the AIF content should lead to increased tAIF content within mitochondria. However, the tAIF content was also decreased in WT following ischemia–reperfusion compared to time control (Fig. 1k). The content of tAIF in cytosol was significantly increased in WT hearts following ischemia–reperfusion compared to time control (Fig. 1m,s). This result suggests that a portion of the tAIF was already released from mitochondria into cytosol in WT during ischemia–reperfusion. The contents of both AIF and tAIF were not markedly altered in deletion mice following ischemia–reperfusion (Fig. 1h,j,l). The content of tAIF in cytosol was not dramatically changed in deletion mouse hearts following ischemia–reperfusion compared to time control (Fig. 1n,t). These results further indirectly support that elimination of CPNS1 prevents the activation of cytosolic CPN1/2 and mitochondrial CPN1 during ischemia–reperfusion.

### CPNS1 deletion decreased cardiac injury during reperfusion

**Figure 2:**
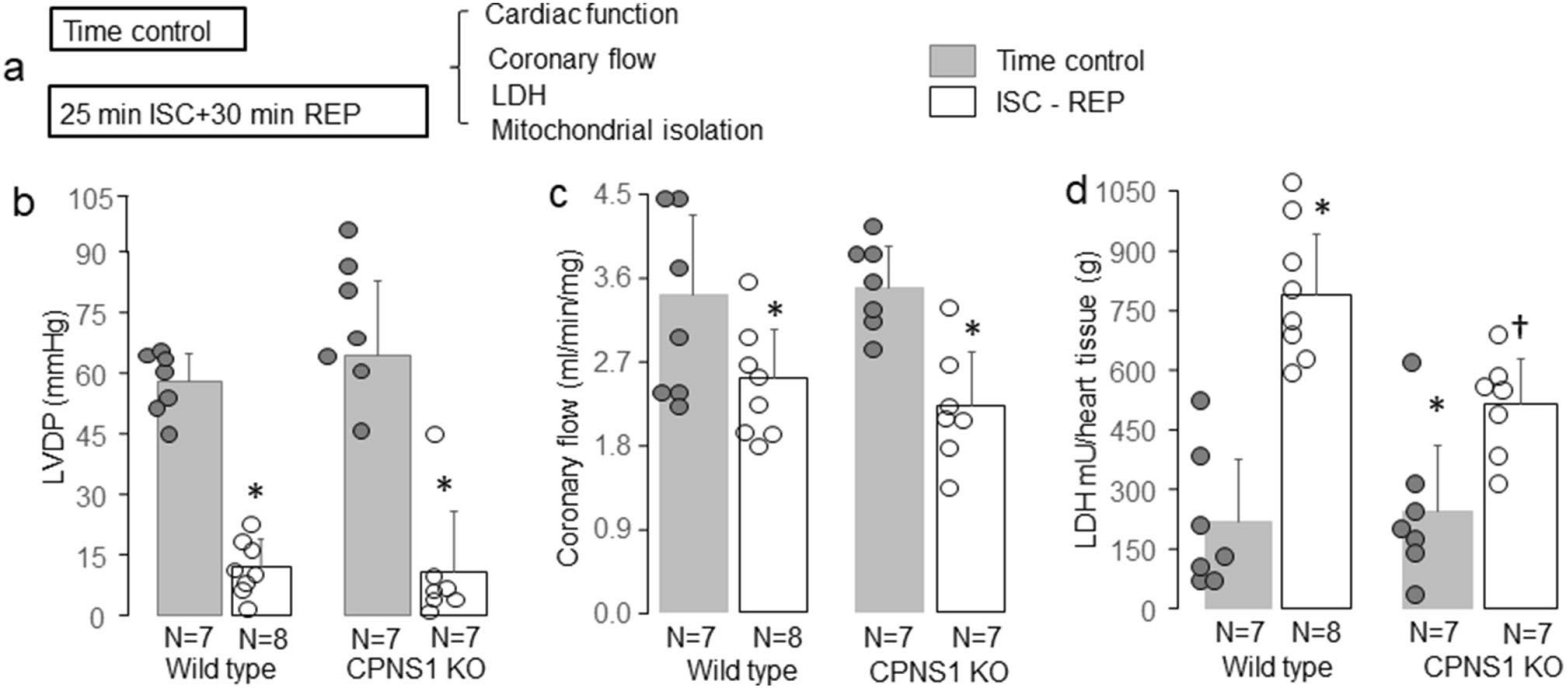
Elimination of CPNS1 decreases cardiac injury during ISC–REP. WT or CPNS1 deletion mouse hearts underwent 25 min global ischemia and 30 min reperfusion. Mouse hearts in time control groups underwent buffer perfusion without ischemia (**a**). ISC–REP led to decreased left ventricular developed pressure (LVDP) in both WT and deletion mice compared to time control (**b**). ISC–REP led to decreased coronary flow in both WT and deletion mice compared to time control (**c**). ISC–REP also increased the release of LDH into the coronary effluent in both WT and deletion compared to time control (**d**). However, LDH release was decreased in CPNS1 deletion mice compared to WT during reperfusion (**d**). These results supported that deletion of CPNS1 decreased cardiac injury during ISC–REP. Data are expressed as mean ± SD; *p < 0.05 vs. time control; ^†^p < 0.05 vs. corresponding WT.

WT or CPNS1 deletion mouse hearts underwent 25 min global ischemia and 30 min reperfusion. Time control hearts were buffer-perfused without ischemia (Fig. 2a). There were no differences in left ventricular developed pressure (LVDP) before ischemia between WT and deletion groups (WT, 58 ± 7 mmHg; deletion 57 ± 9 mmHg, p = NS). Ischemia–reperfusion led to decreased LVDP in both WT and CPNS1 deletion compared to the corresponding time control hearts (Fig. 2b). Compared to WT, deletion of CPNS1 did not improve the recovery of LVDP during reperfusion (Fig. 2b). There were no differences in coronary flow before ischemia between WT and deletion groups (Fig. 2c). Ischemia–reperfusion led to decreased coronary flow in both WT and CPNS1 deletion compared to the corresponding time control hearts (Fig. 2c). Compared to WT, deletion of CPNS1 did not improve the coronary flow during reperfusion (Fig. 2c). There were no differences in LDH release from coronary effluent in time control hearts between WT and CPNS1 deletion (Fig. 2d). Ischemia–reperfusion increased LDH content in the coronary effluent in both WT and CPNS1 deletion mice compared to their corresponding time control (Fig. 2d). The LDH content in coronary effluent in CPNS1 deletion (516 ± 111 mU/g) was significantly lower compared to WT (789 ± 150 mU/g) during reperfusion (Fig. 2d).

**Figure 3:**
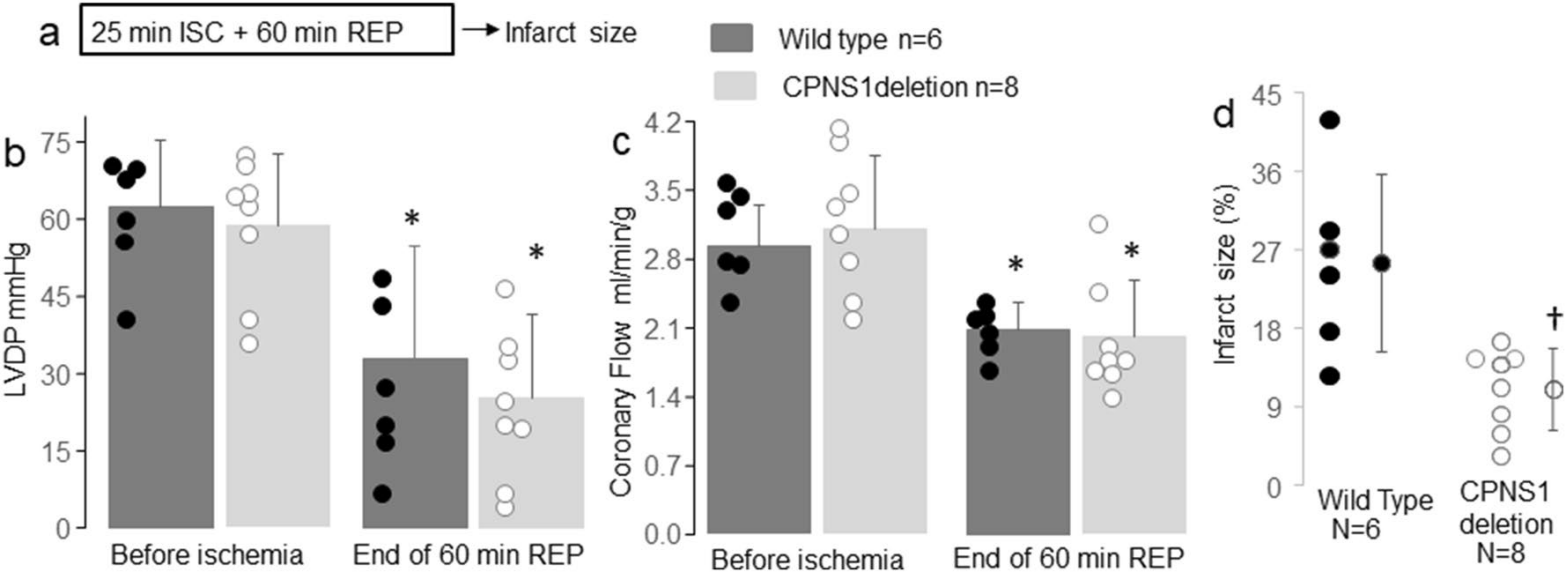
Elimination of CPNS1 decreases infarct size during ISC–REP. In this experiment, WT or CPNS1 deletion mouse hearts underwent 25 min global ischemia and 60 min reperfusion. Infarct size was determined in hearts following 60 min reperfusion using TTC staining. ISC–REP led to decreased left ventricular developed pressure (LVDP) in both WT and deletion mice compared to pre-ischemic LVDP, respectively (**b**). ISC–REP led to decreased coronary flow in both WT and deletion mice compared to pre-ischemic coronary flow, respectively (**c**). Deletion of CPNS1 decreased infarct size during ISC–REP compared to WT (**d**). These results further supported that deletion of CPNS1 decreased cardiac injury during ISC–REP. Data are expressed as mean ± SD; *p < 0.05 vs. time control; ^†^p < 0.05 vs. corresponding WT.

Infarct size was determined in WT or CPNS1 deletion mouse hearts following 25 min ischemia and 60 min reperfusion (Fig. 3a) in separate cohorts of mice. The LVDP was decreased in both WT and deletion mice following 60 min reperfusion compared to pre-ischemic LVDP, respectively (Fig. 3b). There were no differences in coronary flow before ischemia between WT and deletion groups (Fig. 3c). Ischemia–reperfusion led to decreased coronary flow in both WT and CPNS1 deletion compared to the pre-ischemic coronary flow (Fig. 3c). Myocardial infarct size was decreased in CPNS1 deletion (10.9 ± 4.7%) following reperfusion compared to WT (25.4 ± 10.2%) (Fig. 3d). These results further support that deletion of CPNS1 leads to decreased cardiac injury by inhibiting cytosolic and mitochondrial calpains during ischemia–reperfusion.

### CPNS1 deletion improved mitochondrial respiration in hearts measured after reperfusion

**Figure 4:**
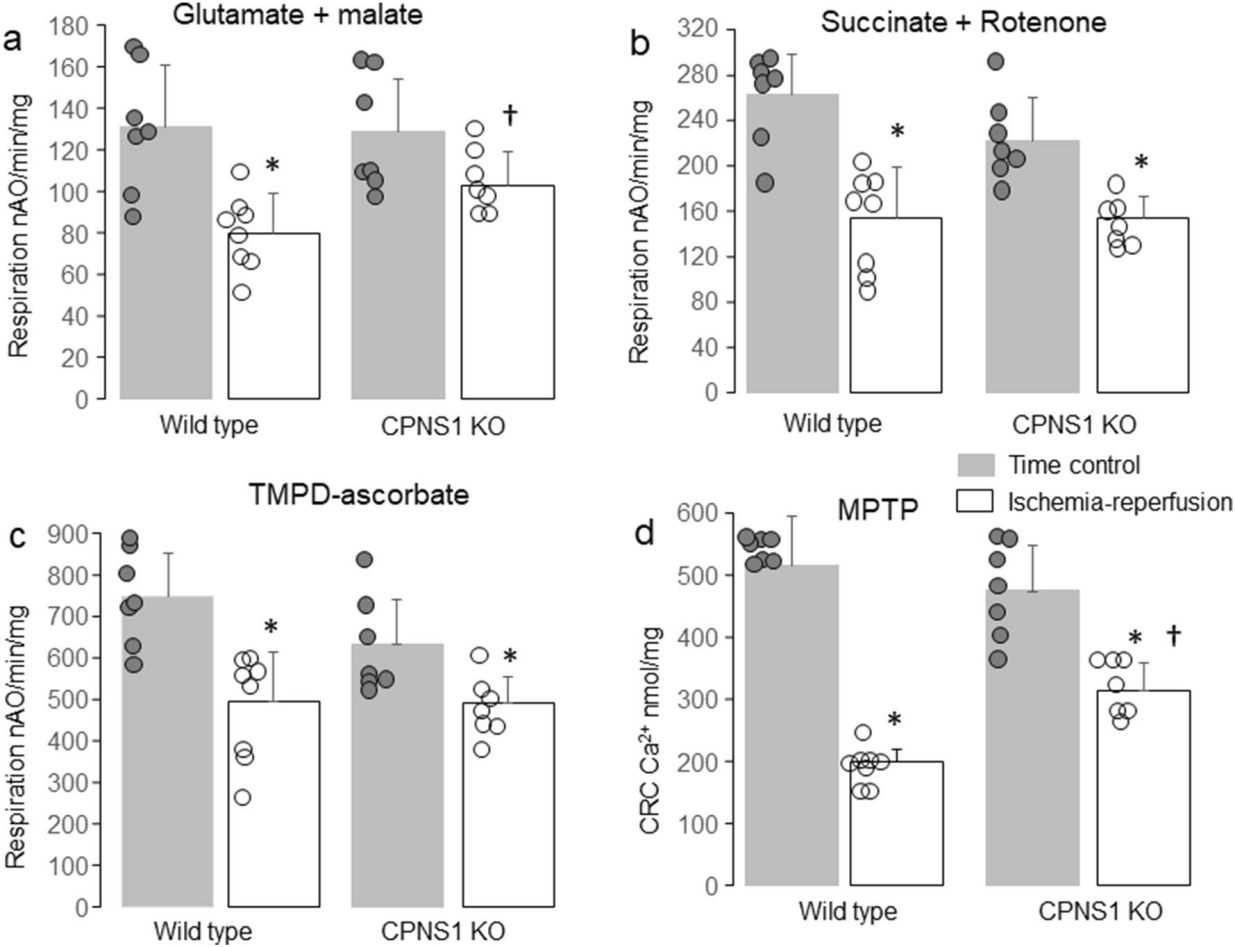
Elimination of CPNS1 improves oxidative phosphorylation and decreases susceptibility to MPTP opening following ISC–REP. Compared to time control, ISC–REP decreased the rate of oxidative phosphorylation in both WT and KO (knockout) using glutamate + malate as complex I substrates (**a**). However, the extent of the decrease in oxidative phosphorylation was less in KO mitochondria following ISC–REP compared to WT (**a**), suggesting that elimination of CPNS1 attenuated complex I damage during ISC–REP. The ISC–REP led to decreased oxidative phosphorylation in both WT and KO using succinate and TMPD-ascorbate as complex II (**b**) and complex IV (**c**) substrates, respectively. Compared to time control, ISC–REP decreased the CRC in both WT and KO (**d**). However, the magnitude of the decrease in CRC in KO was less than that in WT (**d**), suggesting that elimination of CPNS1 inhibited MPTP opening after ischemia–reperfusion injury. Data are expressed as mean ± SD; *p < 0.05 vs. respective time control; ^†^p < 0.05 vs. corresponding WT. N = 7 in each group.

There were no differences in the rate of oxidative phosphorylation in mitochondria from time control hearts between WT and deletion when glutamate + malate, succinate + rotenone, or TMPD-ascorbate + rotenone were used as complex I, II, and IV substrates, respectively (Fig. 4a–c). In WT mice, ischemia–reperfusion decreased the rate of oxidative phosphorylation (80 ± 19 nAO/min/mg) compared to time control using complex I substrate (131 ± 30 nAO/min/mg) (Fig. 4a). However, the rate of oxidative phosphorylation was not markedly altered in CPNS1 deletion mice following ischemia–reperfusion (103 ± 17 nAO/min/mg) with complex I substrate compared to the corresponding time control (129 ± 26 nAO/min/mg). The rate of oxidative phosphorylation in deletion mitochondria following ischemia–reperfusion was higher than that in WT mitochondria in the presence of complex I substrates (Fig. 4a). Ischemia–reperfusion decreased oxidative phosphorylation in mitochondria from both WT and deletion when succinate and rotenone was used as complex II substrate (Fig. 4b). The rate of TMPD-ascorbate oxidation (Fig. 4c) was also decreased in mitochondria from both WT and deletion following ischemia–reperfusion. These results suggest that deletion of CPNS1 attenuates mitochondrial respiratory chain damage, especially in complex I during ischemia–reperfusion.

### CPNS1 deletion decreased MPTP opening in mitochondria isolated after reperfusion

Exogenous calcium was used to trigger MPTP opening in isolated mitochondria. There were no differences in the calcium retention capacity (CRC) between WT (514 ± 81 Ca^2+^ nmol/mg) and deletion (474 ± 72 Ca^2+^ nmol/mg) mitochondria from time control hearts (Fig. 4d). Ischemia–reperfusion decreased the CRC in both WT (200 ± 20 Ca^2+^ nmol/mg) and deletion (314 ± 45 Ca^2+^ nmol/mg) compared to the corresponding time control (Fig. 4d). The CRC was significantly greater in CPNS1 deletion following reperfusion compared to WT (Fig. 4d), indicating that deletion of CPNS1 decreases the susceptibility to MPTP opening following ischemia–reperfusion injury.

### CPNS1 deletion decreased ROS generation in mitochondria isolated after reperfusion

There were no differences in the net release of H_2_O_2_ from intact mitochondria between WT and deletion with or without ischemia–reperfusion when glutamate + malate was used as complex I substrate (Table 1). Since mitochondria include endogenous antioxidants, some of the H_2_O_2_ generated within intact mitochondria are detoxified by mitochondrial antioxidants. In contrast, mitochondrial antioxidants are released from the matrix of detergent solubilized mitochondria. Thus, the permeabilized mitochondria can be used to assess the total H_2_O_2_ generation. NADH is unable to pass through the inner mitochondrial membrane in intact mitochondria. However, NADH can traverse the inner membrane in the permeabilized mitochondria. Thus, NADH is used as a complex I substrate in the solubilized mitochondria. There was no difference in the total H_2_O_2_ generation from solubilized mitochondria between WT and deletion time control when NADH was used as complex I substrate (Table 2). However, ischemia–reperfusion led to increased total H_2_O_2_ generation in permeabilized WT mitochondria compared to deletion using NADH as complex I substrate (Table 1).

**Table 1:** The rate of H_2_O_2_ generation in subsarcolemmal mitochondria (SSM) from wild type and CPNS1 deletion with or without ISC–REP.

Mice	Wild type		CPNS1 deletion	
Groups	Time control (n = 7)	ISC–REP (n = 8)	Time control (n = 7)	ISC–REP (n = 7)
Intact mitochondria	Complex I substrates: Glutamate + Malate			
H_2_O_2_ (pmol/min/mg)	84 ± 19	75 ± 22	80 ± 16	82 ± 24
	Complex II substrates: Succinate			
H_2_O_2_ (pmol/min/mg)	974 ± 419	280 ± 94*	437 ± 165 *	172 ± 62^†^
	Complex II substrates: Succinate + Rotenone			
H_2_O_2_ (pmol/min/mg)	405 ± 63	394 ± 127	282 ± 98*	240 ± 77^‡^
	Reverse flow-induced ROS generation			
H_2_O_2_ (pmol/min/mg)	678 ± 357	–	148 ± 120*	–
Solubilized Mitochondria	Complex I substrate: NADH			
H_2_O_2_ (pmol/min/mg)	752 ± 63	1029 ± 214*	608 ± 160	630 ± 59^‡^

Mean ± SD. *p < 0.05 vs. corresponding WT time control. ^†^p < 0.05 vs. CPNS1 deletion time control. ^‡^p < 0.05 vs. wild type ISC–REP. Reverse flow-induced ROS represents the difference between the rate of H_2_O_2_ generation using succinate alone as substrate and the rate of H_2_O_2_ generation using succinate as substrate in the presence of rotenone to block reverse electron flow.

**Table 2:** Deletion of CPNS1 alters mitochondrial (IFM) proteins in ischemia–reperfusion hearts (IR, deletion/WT).

Protein	Accession	Gene	LFQ ratios	p-value	Expression
		ID	IR deletion/WT		
Succinate dehydrogenase assembly factor 2, mitochondrial	Q8C6I2	Sdhaf2	0.47	0.0268	↓ in deletion IR
39S ribosomal protein L43, mitochondrial	Q99N89	Mrpl43	0.27	0.0037	↓ in deletion IR
Cytochrome c-type heme lyase	P53702	Hccs	2.41	0.0408	↑ in deletion IR
Paraplegin	Q3ULF4	Spg7	2.08	0.0052	↑ in deletion IR

Reverse electron flow-induced ROS represents the difference between the rate of H_2_O_2_ generation using succinate alone as substrate and the rate of H_2_O_2_ generation using succinate as substrate in the presence of rotenone to block reverse electron flow^34^. In time control hearts, H_2_O_2_ generation from reverse electron flow was significantly decreased in deletion compared to WT when succinate was used as complex II substrate (Table 1). The reverse flow-induced ROS generation was eliminated in both WT and deletion mitochondria following ischemia–reperfusion injury compared to the corresponding time control (Table 1). Deletion of CPNS1 produced less H_2_O_2_ generation following ischemia–reperfusion injury compared to WT when succinate + rotenone was used as substrate (Table 1).

### CPNS1 deletion decreased cytochrome c release and PARP activation following reperfusion

**Figure 5:**
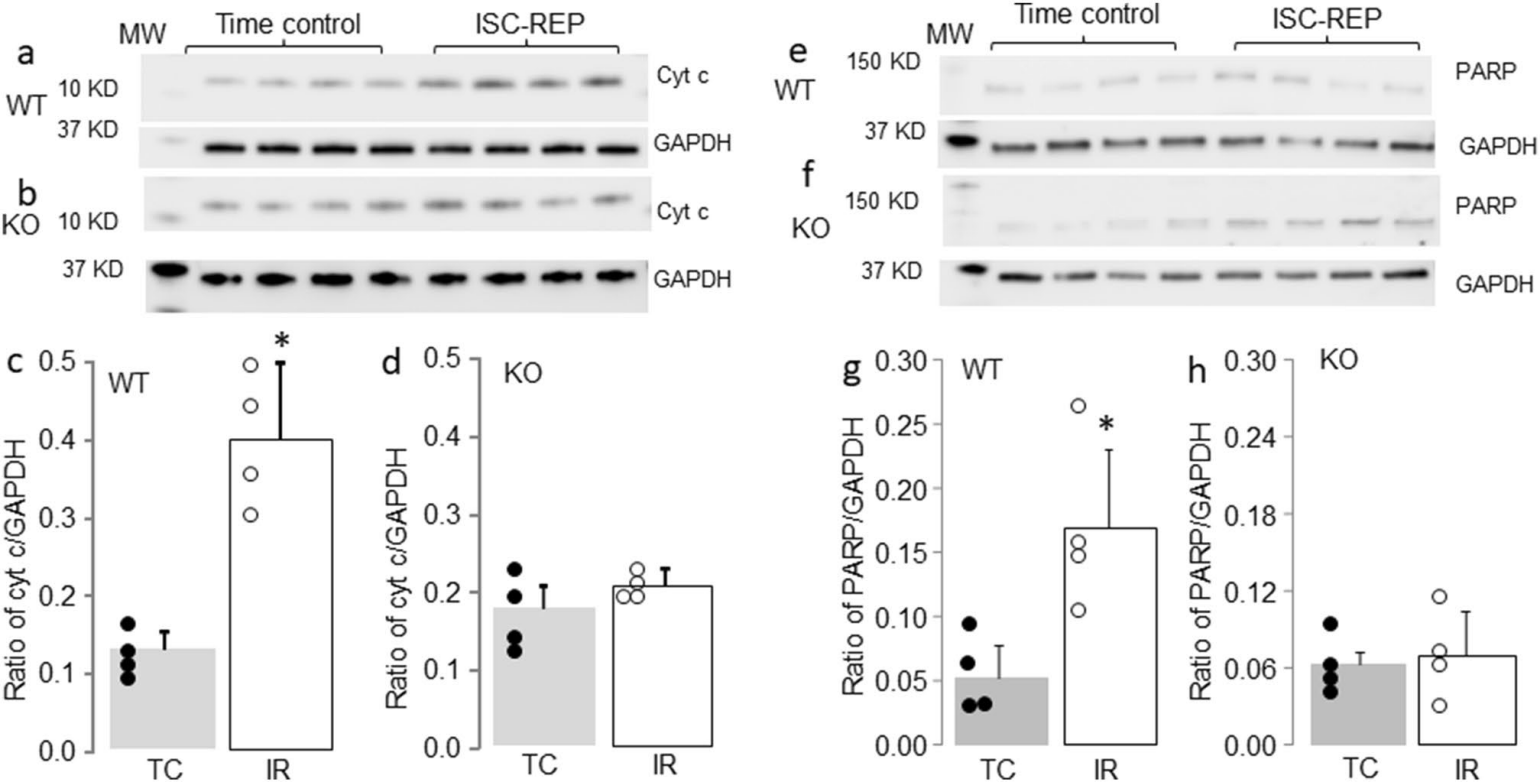
Elimination of CPNS1 decreases cytochrome *c* loss and PARP activation during ISC–REP. In wild type mice, ISC–REP led to increased cytochrome *c* content in cytosol compared to time control (**a**,**c**). However, ISC–REP did not increase cytochrome *c* content in cytosol in KO (knockout) mice compared to time control (**b**,**d**). These results suggest that elimination of CPNS1 maintained mitochondrial outer membrane integrity during ISC–REP. In wild type mice, ISC–REP led to increased PARP cleavage compared to time control (**e**,**g**). However, ISC–REP did not increase PARP cleavage in KO mice compared to time control (**f**,**h**), indicating that elimination of CPNS1 prevented AIF-mediated PARP cleavage during ISC–REP. Data are expressed as mean ± SD; *p < 0.05 vs. time control. n = 4 in each group. Not every sample was used for immunoblotting in each group.

Compared to time control, the cytochrome *c* content in cytosol was increased in WT mice following ischemia–reperfusion injury (Fig. 5a,c). Deletion of CPNS1 substantially decreased the release of cytochrome *c* from mitochondria into cytosol after ischemia–reperfusion (Fig. 5b,d). Ischemia–reperfusion increased the content of cleaved PARP (90 KD) in WT compared to time control (Fig. 5e,g). Deletion of CPNS1 decreased the content of cleaved PARP after ischemia–reperfusion injury (Fig. 5f,h). A release of the tAIF from mitochondria into cytosol contributes to the activation of caspase-independent apoptosis through PARP cleavage and activation^18^. Deletion of CPNS1 prevents PARP activation after ischemia–reperfusion, suggesting that the decreased tAIF release from mitochondria in deletion mice attenuates PARP activation and subsequent cardiac injury following ischemia–reperfusion.

### Ischemia–reperfusion altered the mitochondrial proteome in wild type mice

**Figure 6:**
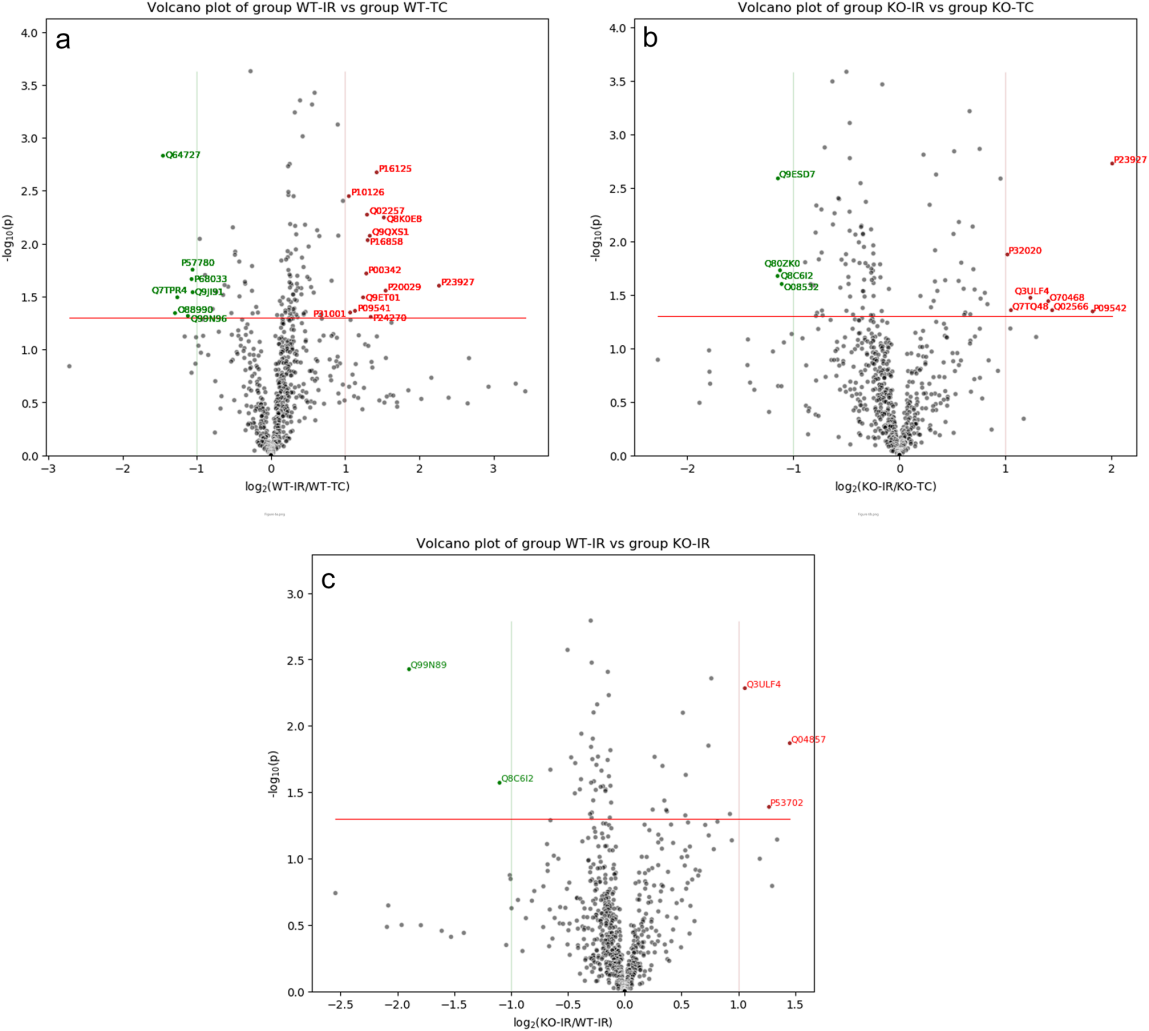
Analysis of proteomic data with volcano-plot in WT and CPNS1 KO (knockout) mitochondria. (**a**) Volcano-plot of WT-IR vs WT-TC. (**b**) Volcano-plot of KO-IR vs KO-TC. (**c**) Volcano-plot of KO-IR vs WT-IR. The ratios of the average protein abundances in WT-IR group to the average protein abundances in WT-TC group were presented in log2() scale on the x-axis, and the *p* value from t-test between these 2 groups were presented in − log10() scale on the y-axis. The significant threshold for *p*-value was set at < 0.05 (red line). Above this threshold, the proteins with greater than twofold increased abundances in WT-IR group over WT-TC group were colored in red, and the proteins with greater than twofold decreased abundances in WT-IR group over WT-TC group were colored in green.

Quantitative proteomics was performed on isolated heart mitochondria (IFM) from WT time control and WT ischemia–reperfusion mice. IFM were used in the current study due to less cytosolic contamination^22^. This proteomic analysis identified a total of 692 proteins and the relative abundance of these proteins was compared using Label free quantitation (Fig. 6a). This comparison identified 13 proteins that are more abundant (> two-fold, *p* value < 0.05) in the WT- ischemia–reperfusion mitochondria including α-crystallin B chain, endoplasmic reticulum chaperone BiP, and catalase (Table 3). A total of 7 proteins are less abundant in the ischemia–reperfusion WT mitochondria (< two-fold, *p* value < 0.05) including tetratricopeptide repeat protein 19 and 39S ribosomal protein L1 (mitochondrial) (Table 3).

**Table 3:** Ischemia–reperfusion alters mitochondrial (IFM) protein in wild type mice (WT:IR/TC).

Protein	Accession	Gene	LFQ ratios	p-value	Expression
		ID	WT IR/TC		
Tetratricopeptide repeat protein 19	Q8CC21	Ttc19	0.4	0.00052	↓ in WT IR
39S ribosomal protein L1, mitochondrial	Q99N96	Mrpl1	0.46	0.0482	↓ in WT IR
Alpha-crystallin B chain	P23927	Cryab	4.81	0.0250	↑ in WT IR
Endoplasmic reticulum chaperone BiP	P20029	Hspa5	2.92	0.0278	↑ in WT IR
Catalase	P24270	Cat	2.54	0.0492	↑ in WT IR

The relationship between several differentially expressed proteins and reperfusion injury of hearts in wild type mice was generated using the Pathway Explorer tool in Qiagen Ingenuity Pathway Analysis (IPA). The predictions were made by Molecule Activity Predictor (MAP) of IPA based on the mitochondrial protein content ratios of IR/TC in wild type mice and the *p* values (Fig. 6a).

Upregulated CRYAB in WT-IR could be a result of inhibited Mek. The elevated level of catalase could be caused by down-regulated PRKAA, c-Jun N-terminal kinase (JNK), and NFκB (complex). Down-regulated NFκB (complex) could also activate desmin. The predicted inhibition of ascorbic acid (Vitamin C) could be the reason of upregulated heat shock protein family A (Hsp70) member 5 (HSPA5), which predicts activation of calpain. This agrees with our hypothesis of cytosolic and mitochondrial calpain activation during ischemia–reperfusion.

### Ischemia–reperfusion altered mitochondrial proteins in CPNS1 deletion mice

The quantitative proteomic comparison of heart mitochondria from deletion time control and deletion ischemia–reperfusion resulted in the identification of 692 proteins (see Fig. 6b, Volcano plot). A total of 7 proteins were identified to be more abundant in the deletion ischemia–reperfusion hearts (> two-fold, *p* value < 0.05) including α-crystallin B chain, paraplegin, and sarcalumenin (Table 4). Among these up-regulated proteins, paraplegin, an ATP-dependent zinc metalloprotease, plays a role in assembling and regulating the mitochondrial permeability transition pore (MPTP)^35^.

**Table 4:** Ischemia–reperfusion alters mitochondrial (IFM) protein in CPNS1 deletion mice (Deletion:IR/TC).

Protein	Accession	Gene	LFQ ratios	p-value	Expression
		ID	Deletion IR/TC		
28S ribosomal protein S10, mitochondrial	Q80ZK0	Mrps10	0.46	0.0187	↓ in deletion IR
Succinate dehydrogenase assembly factor 2, mitochondrial	Q8C6I2	Sdhaf2	0.45	0.0211	↓ in deletion IR
Alpha-crystallin B chain	P23927	Cryab	4.02	0.0019	↑ in deletion IR
Paraplegin	Q3ULF4	Spg7	2.35	0.0337	↑ in deletion IR
Sarcalumenin	Q7TQ48	Srl	2.07	0.0440	↑ in deletion IR

**Figure 7:**
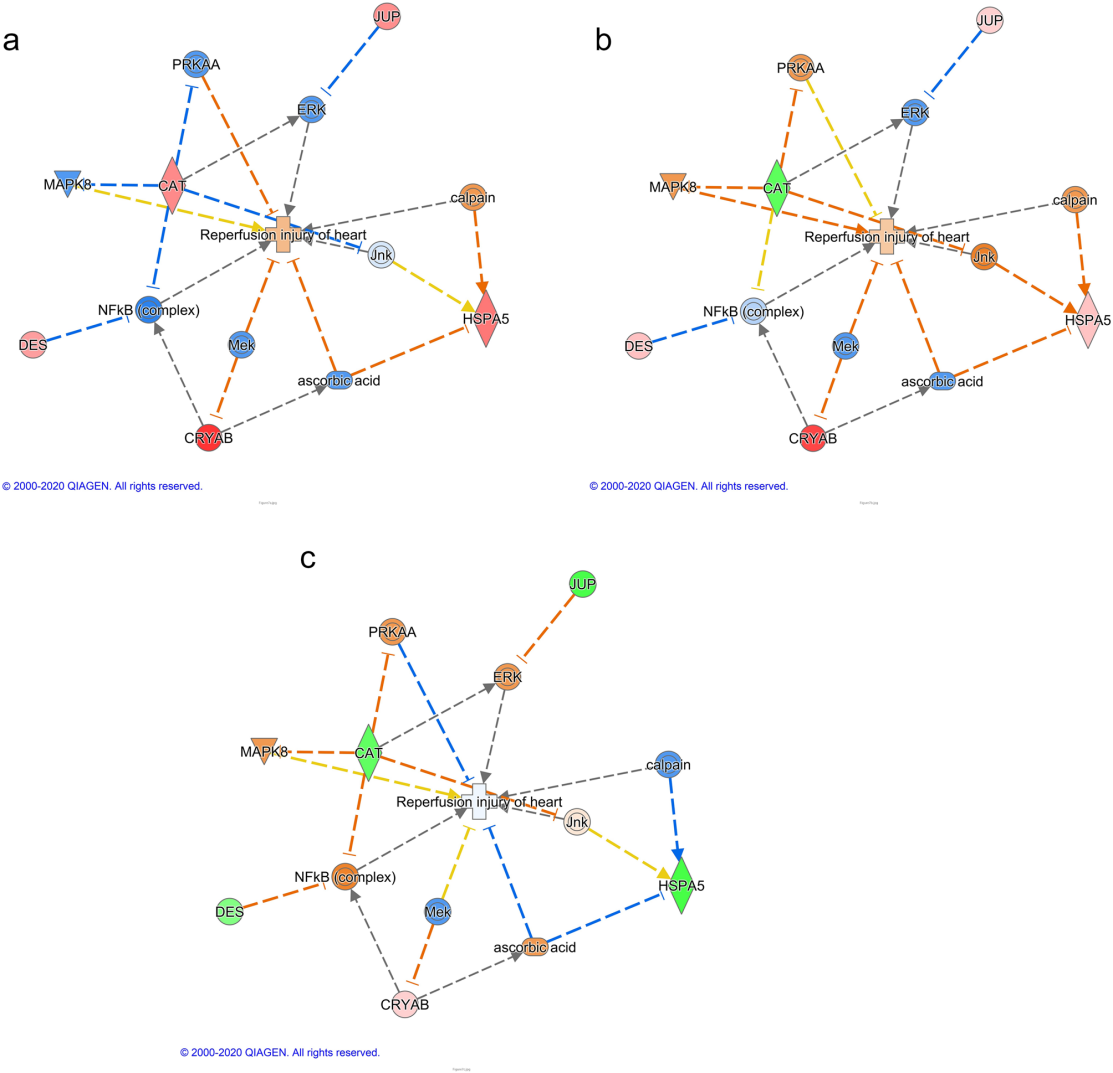
Analysis of proteomic data with Ingenuity Pathway Analysis (IPA) in WT and CPNS1 KO (knockout) mitochondria. (**a**) Predicted effects using WT:IR/TC data in the proteins-IR network. (**b**) Predicted effects using KO:IR/TC data in the proteins-IR network. (**c**) Predicted effects using IR: KO/WT data in the proteins-IR network. The figure was generated in Qiagen Ingenuity Pathway Analysis (IPA) program using several differentially expressed proteins (pink or red for upregulated protein, green for downregulated proteins) and their relationships to reperfusion injury of heart. The relationships and several mediating proteins, complexes or endogenous chemicals were added using the Pathway Explorer tool in IPA, and the predicted effects (orange for activation, blue for inhibition, yellow indicates the finding is inconsistent with the state of downstream molecule, and grey for no prediction) were made by the Molecule Activity Predictor (MAP) of IPA. Relationships:  Direct interaction.  Indirect interaction.  activation, causation, expression, localization, membership, modification, phosphorylation, regulation of binding, transcription.  inhibition, ubiquitination.

A total of 4 proteins were identified to be less abundant in the deletion ischemia–reperfusion hearts (> two-fold, *p* value < 0.05) including 28S ribosomal protein S10 (mitochondrial), and succinate dehydrogenase assembly factor 2 [mitochondrial (SDHF2)] (Table 3). SDHF2 is essential in assembling succinate dehydrogenase (SDH), a component of complex II, and the latter is a component of both the TCA cycle and the ETC^6^. The relationship between several differentially expressed proteins and reperfusion injury of hearts in deletion mice was generated using the same pathway as in Fig. 7a, and the predictions were made by Molecule Activity Predictor (MAP) of IPA based on the mitochondrial protein content ratios of IR/TC in CPNS1 deletion mice and the *p* values (Fig. 6b).

There was a similar pattern in this data set as in Fig. 7a except catalase was downregulated. The decreased level of catalase could be a result of activated PRKAA, JNK and MAPK8. The predicted inhibition of ascorbic acid (Vitamin C) and activated JNK could be the reason for up-regulated HSPA5, which predicted activation of calpain. This figure also displayed calpain activation during ischemia–reperfusion.

### CPNS1 deletion altered proteins in ischemia–reperfusion hearts

The quantitative proteomic comparison of heart mitochondria from WT ischemia–reperfusion and deletion ischemia–reperfusion resulted in the identification of 3 proteins (Fig. 6c). A total of 3 proteins were identified to be more abundant in deletion ischemia–reperfusion mitochondria (> two-fold, *p* value < 0.05) including cytochrome *c*-type heme lyase^36^ and paraplegin (Table 4). Paraplegin showed an increased abundance in both deletion-IR vs. deletion-TC comparison and deletion-IR (Fig. 6b) vs. WT-IR comparison (Fig. 6c) suggesting both the deletion of CPNS1 and ischemia–reperfusion contributed to the change of paraplegin.

A total of 2 proteins were identified to be less abundant in the deletion ischemia–reperfusion hearts (> two-fold, *p* value < 0.05) including succinate dehydrogenase assembly factor 2 (SDHF2) and 39S ribosomal protein L43 (mitochondrial) (Table 2). Protein SDHF2 showed a decrease in both deletion-ischemia–reperfusion (IR) vs. deletion-time control (TC) comparison and deletion-IR vs. WT-IR comparison suggesting both the deletion of CPNS1 and ischemia–reperfusion contributed to the change of SDHF. The relationship between several differentially expressed proteins and reperfusion injury of hearts between WT and deletion mice using the same pathway as in Fig. 7a was used. The predictions were made by MAP of IPA based on the mitochondrial protein content ratios of deletion/WT in mouse hearts following ischemia–reperfusion and the *p* values (Fig. 7c).

This figure (Fig. 7b) predicted an inhibition of reperfusion injury of hearts in IR-deletion. Lower level of catalase could be caused by activated PRKAA and NFκB (complex). Activated NFκB (complex) was predicted by decreased desmin. Activated ascorbic acid was predicted by decreased HSPA5, which in turn inhibits ischemia–reperfusion injury. Downregulation of HSPA5 could be a result of a decrease in the activity of calpain. This figure suggests decreased that a calpain content and activity inhibits reperfusion injury of hearts.

## Discussion

The pharmacologic inhibition of calpain1/2 decreases cardiac injury during ischemia–reperfusion^2,3,15^. Upregulation of mitochondria-localized CPN1 facilitates the development of heart failure^1^. Inhibition of CPN1/2 by overexpressing the endogenous inhibitor-calpastatin decreases cardiac injury during ischemia–reperfusion^37^, though the predominant impact in this study is predicted to be on cytosolic calpain activation due to the localization of calpastatin in the cytosol. In the present study, we show that cardiomyocyte specific CPNS1 deletion decreases cardiac injury following ischemia–reperfusion (Figs. 2, 3), supporting that activation of CPN1/2 contributes to cardiac injury during ischemia–reperfusion. The mechanisms of protection in CPNS1 deletion mice involve less ROS generation and a decreased sensitivity to MPTP opening. Deletion of CPNS1 preserved proteins including paraplegin and sarcalumenin that play key roles in regulating mitochondrial calcium homeostasis. Inhibition of CPN1/2 also preserved proteins including tetratricopeptide repeat protein 19 that is essential for complex III assembly. These results support that the deletion of CPNS1 decreases cardiac injury during reperfusion by improving mitochondrial function.

Ischemia–reperfusion leads to mitochondrial dysfunction by impairing the ETC^6,38–41^. Ischemia–reperfusion leads to decreased activities of complex I^3,38^, complex III^6^, and complex IV^6^. In wild type mice, proteomic data shows that ischemia–reperfusion leads to a decreased content of 39S ribosomal protein L1 (MRPL1) (Table 3). MRPL1 is a mitochondrial ribosomal protein that assists mitochondrial protein synthesis of mtDNA encoded large subunits^42^. The mtDNA encoded subunits include complex I subunits (ND1, ND2, ND3, ND4, ND4L, ND5, and ND6)^43^, a complex III subunit (cytochrome *b*)^44^, and complex IV subunits (subunits I, II, and III)^45^. The current study indicates a decrease in MRPL1 content may contribute to the ETC defect by impairing the synthesis of mtDNA-encoded subunits. Tetratricopeptide repeat protein 19 is important to maintain complex III activity by regulating the turnover of the Rieske iron-sulfur protein^46^. Complex III activity is decreased in cardiac mitochondria following ischemia due to dysfunction of the Rieske protein^47^. The current study suggests that ischemia–reperfusion decreases complex III activity by impairing the function of the Rieske protein perhaps through altering tetratricopeptide repeat protein 19 content (Table 3).

Ischemia–reperfusion also leads to increased contents of α-crystallin B chain, BiP, and catalase in WT (Table 3). The α-crystallin B chain belongs to the small heat shock protein family^48,49^, and its function is to prevent protein aggregation by binding with misfolded proteins^50^. BiP (GRP78) is an HSP70 molecular chaperone that exists in the endoplasmic reticulum (ER), and its function is involved in protein folding and assembly^51^. Although GRP78 is an ER chaperone, it is also present at the mitochondria-associated membranes (MAM). The GRP78 plays a critical role in protein folding including in the MAM^52^. An increase in GRP78 expression also leads to decreased Mitofusin 2 (MFN2)^53^ content that is critical to tether ER and mitochondrial together^54^. Ischemia–reperfusion increases the ER stress that leads to accumulation of misfolded proteins^55^. Ischemia–reperfusion also increases the ROS generation in cardiac mitochondria^38^. Catalase is an antioxidant enzyme that scavenges the H_2_O_2_. These results indicate that adaptive reactions to repair mitochondrial function occur during ischemia–reperfusion in wild type mice.

In CPNS1 deletion mice, ischemia–reperfusion leads to an increased content of the α-crystallin B chain, paraplegin, and sarcalumenin (Table 4). Paraplegin is a mitochondrial protease located in the inner mitochondrial membrane^56^. Paraplegin plays a key role in mitochondrial protein quality control. Mutation of the paraplegin gene leads to impaired mitochondrial function and a neurodegenerative disorder^56^. Sarcalumenin is a calcium-binding protein that regulates calcium homeostasis within the ER^57^. The deficiency of sarcalumenin disrupts calcium homeostasis in sarcoplasmic reticulum (SR) that impairs cardiac function^57^. SR/ER are connected with mitochondria through MAM^58^. Alteration of the intracellular calcium level will lead to mitochondrial dysfunction by affecting mitochondrial calcium homeostasis^6,22,59^. Thus, deletion of CPNS1 may provide a beneficial effect by maintaining mitochondrial calcium homeostasis through preservation of the paraplegin and sarcalumenin contents. Cytochrome *c*-type heme lyase contributes a key role in attaching the heme group to the apoprotein of cytochrome *c*^60^. In WT mice, ischemia–reperfusion leads to the loss of cytochrome *c* from mitochondria (Fig. 5). Deletion of CPNS1 decreased the loss of cytochrome *c* during reperfusion (Fig. 5). In addition, deletion of CPNS1 also protects cytochrome *c*-type heme lyase content during ischemia–reperfusion. These results suggest that deletion of CPNS1 not only preserves cytochrome *c* content, but also maintains its function in mitochondria following ischemia–reperfusion as a potential mechanism of the preserved function of electron transport.

Deletion of CPNS1 leads to decreased succinate dehydrogenase assembly factor 2 (SDHAF2) (Table 4) that is essential for the assembly of succinate dehydrogenase^61^. These decreased proteins may explain why deletion of CPNS1 does not improve OXPHOS with succinate as a complex II substrate in mitochondria during ischemia–reperfusion.

Ischemia–reperfusion leads to markedly decreased oxidative phosphorylation in WT oxidizing complex I, II, and IV substrates compared to time control. In contrast, ischemia–reperfusion does not decrease oxidative phosphorylation in deletion mice with complex I substrates (Fig. 4). Oxidation of succinate and TMPD-ascorbate is still decreased in deletion mice following ischemia–reperfusion. These results suggest that deletion of CPNS1 leads to less damage in complex I during reperfusion. This result provides mechanistic validation of the previous findings that administration of calpain inhibitors protect complex I in cardiac mitochondria following ischemia–reperfusion^3,16^. Proteomic study shows that ischemia–reperfusion leads to decreased succinate dehydrogenase assembly factor 2 (SDHAF2) (Table 2) that is critical for complex II assembly^62^. A decrease in SDHAF2 may contribute to the complex II defect in deletion mice during reperfusion^62^ but may also serve to attenuate the deleterious reverse electron flow from complex II to complex I that occurs during early reperfusion^63,64^.

The respiratory chain is a key source of ROS generation in cardiac mitochondria^65,66^. Complex I and complex III are major sites of ROS generation^65,66^. ROS can be generated by forward electron flow using complex I substrates or reverse electron flow with complex II substrate (succinate)^34,63,64^. In time control hearts, deletion of CPNS1 does not alter ROS generation with complex I substrates compared to WT, indicating that CPNS1 deletion does not alter forward-electron flow-induced ROS generation (Table 1). However, deletion of CPNS1 dramatically decreases ROS generation with succinate as substrate, indicating that CPNS1 deletion decreases the reverse electron flow-induced ROS generation. The decreased reverse electron flow-induced ROS generation may be due to a reduction of the SDHAF2 content that limits electron flow into complex II. In addition, deletion of CPNS1 also decreases ROS generation when succinate + rotenone is used as substrate. This latter result indicates that deletion of CPNS1 also decreases ROS generation from complex II^67^ and likely also from complex III^65^ in that the reverse electron flow is blocked by rotenone^34,63,64^. The reverse electron flow-induced ROS generation contributes to cardiac injury during ischemia–reperfusion^64^. A decrease in the reverse flow-induced ROS generation should decrease cardiac injury during reperfusion. The reverse flow-induced ROS generation is sensitive to depolarization of inner mitochondrial membrane potential^63,68^. Ischemia–reperfusion leads to decreased inner membrane damage with a decrease in membrane potential observed in isolated cardiac mitochondria^69^. The reverse electron flow-induced ROS generation is also decreased in cardiac mitochondria^34,63^. Our current studies show that the reverse flow-induced ROS generation was decreased in both WT and deletion mice, consistent with previous studies^34,63^. The decreased ROS generation from reverse electron flow after ischemia–reperfusion may be due to the depolarization of inner mitochondrial potential that occurred during reperfusion due to the inner membrane damage^63^.

In freshly isolated mitochondria, only the net release of H_2_O_2_ can be detected because ROS generated within mitochondria are detoxified by mitochondrial antioxidants^65^. Thus, ROS generation was also measured in detergent-solubilized mitochondria. In this condition, mitochondrial antioxidants are markedly diluted in the assay buffer and exert less antioxidant effect. Ischemia–reperfusion increases ROS generation in detergent-solubilized mitochondria from WT with NADH as substrate, supporting that the damaged respiratory chain during ischemia–reperfusion increases total ROS generation (Table 1). Interestingly, ischemia–reperfusion does not increase total ROS generation in CPNS1 deletion mice, supporting that deletion of CPNS1 decreases ROS generation during reperfusion.

MPTP opening is considered as a final step in cell death following reperfusion^19^. Inhibition of MPTP opening using physiologic^70,71^ or pharmacologic approaches^71^ decreased cardiac injury during reperfusion. Genetic ablation of complex I subunits sensitizes to MPTP opening during pressure overload, suggesting that the damaged complex I sensitizes to MPTP opening^72,73^. Activation of CPN2 increases MPTP opening during ischemia–reperfusion^16^. Inhibition of CPN1/2 using MDL-28170 decreases MPTP opening during ischemia–reperfusion^3,15^. The current study shows that deletion of CPNS1 decreases the susceptibility to MPTP opening during ischemia–reperfusion (Fig. 4). These results provide straightforward evidence that activation of CPN1/2 sensitizes to MPTP opening during ischemia–reperfusion. Protection of complex I during ischemia–reperfusion decreases MPTP opening^3,16,69,73^.

The present study has limitations. The activities of CPN1 and 2 were not directly measured with a fluorescence method^2,74^. Since the cleavage of spectrin and AIF are used to reflect cytosolic and mitochondrial CPN1 activation, lack of CPN1/2 activity measurement will not affect the interpretation of the current results. In this study, we used an in vitro rather than in vivo ischemia–reperfusion model to study heart and mitochondrial injury in that two populations of mitochondria can be isolated from single mouse hearts following global ischemia–reperfusion^75,76^. It is a technical challenge to isolate the two populations of mitochondria from the risk area in mouse heart following in vivo ischemia–reperfusion. Since the amount of SSM is limited after use in functional assays, IFM were used for proteomic study. Only 4 samples in each group were used for proteomic analysis. Another advantage to the use of IFM for proteomic study is to decrease potential cytosolic contamination that is removed during the IFM isolation procedure as a result of the trypsin treatment^22^. Our previous study showed that protein level decreases of > 50% or increased > twofold can be verified with immunoblotting^75^. Therefore, we only listed proteins either decreased > 50% or increased > twofold in the current study. The effect of CPNS1 deletion on protein changed in the basal condition needs to be addressed in the future. Thus, most of the protein changes revealed by proteomic study were not verified by immunoblotting.

In our previous studies, we found that prevention of mitochondrial damage during ischemia led to decreased cardiac injury during reperfusion^38,39^. These results support that the mitochondrial dysfunction contributes to cardiac injury during ischemia–reperfusion. In the current study, the genetic downregulation of CPN1/2 decreases cardiac injury by protecting mitochondria, especially complex I driven respiration during ischemia–reperfusion. Inhibition of CPN1/2 not only prevents AIF cleavage (Fig. 1), but also alters several mitochondrial protein targets (Tables 2, 3, 4). The downregulation of CPN1/2 decreased the susceptibility to MPTP opening (Fig. 4), suggesting that mitochondria localized calpains and the MPTP may contribute to a mutually reinforcing cycle of calcium mediated injury during ischemia and reperfusion. Genetic inhibition of CPN1/2 decreased the release of cytochrome *c* (Fig. 5) and tAIF (Fig. 1) to trigger both caspase dependent and caspase independent cell death^18,77^. The identification of potential calpain targets within mitochondria will provide insight into new strategies to protect mitochondria and the myocardium during ischemia–reperfusion.

## Methods

All animal procedures were approved by the Animal Care and Use Committees of the McGuire VA Medical Center and Virginia Commonwealth University and were conducted in accordance with the guidelines provided by the NIH for Animal Care. All methods were performed in accordance with the ARRIVE (Animal Research: Reporting of In Vivo Experiments) guidelines.

### Generation of CPNS1 deletion mice

Cardiac specific CPNS1 deletion mice (CPN4^P/P^) are generated by breeding the floxed CPNS1^PZ/PZ^ mice in C57BL/6 background (provided by Dr. Peter Greer from Queen's University Cancer Research Institute, Kingston, Ontario, Canada^27^) crossed with MHC-MerCreMer mice [B6.FVB(129)-A1cf^Tg(Myh6-cre/Esr1*)1Jmk^/J], which are in C57BL/6 background and have the cardiac-specific and tamoxifen-inducible *cre* recombinase, to generate CAPNS1^PZ/W^.*cre* mice. The CAPNS1^PZ/W^.*cre* mice are back crossed with CAPNS1^PZ/PZ^ to generate CAPNS1^PZ/PZ^.*cre* mice. The CAPNS1 deletion mice were genotyped with PCR primer: P1 (GTC AGG CTA GAT GCC ATG TTC C), P2 (CGA CTA TCC GAG CGC TGC C), and P3 (GTT CAC TTG GAT CTG TCC GGT GCC). The primers used for *cre* detection were: P1 (ATA TCT CAC GTA CTG ACG GTG GG) and P2 (CTG TTT CAC TAT CCA GGT TAG GG). The CAPNS1^PZ/PZ^.*cre* mice are treated with tamoxifen [IP injection (1 mg/day), daily for 4 days]^78^ to generate cardiac specific CAPNS1 deletion mice (CAPNS1^P/P^) mice. The tamoxifen-treated CAPNS1^PZ/PZ^ mice without cre are also treated with tamoxifen and used as control. Mice (2–3 mo. old) received the tamoxifen treatment. Mice were used within 2–3 weeks following tamoxifen treatment. The CPNS1^PZ/PZ^ mice were used as wild type (WT) mice and CPNS1^P/P^ mice were used as deletion mice^27^. There was no difference in ratio of heart/body weight both in time control groups [0.0053 ± 0.0003 (WT, n = 7) vs. 0.0057 ± 0.0004 (deletion, n = 7), p = NS] and in ischemia–reperfusion groups [0.0057 ± 0.0003 (WT, n = 8) vs. 0.0053 ± 0.0003 (deletion, n = 7), p = NS].

### Preparation of mouse hearts for perfusion

WT and CPNS1 deletion mice were anesthetized with pentobarbital sodium (100 mg/kg, i.p.) and anticoagulated with heparin (1000 IU/kg, i.p.). The heart was isolated and retrogradely perfused on a Langendorff apparatus via the aorta with modified Krebs–Henseleit buffer (115 mM NaCl, 4.0 mM KCl, 2.0 mM CaCl_2_, 26 mM NaHCO_3_, 1.1 mM MgSO_4_, 0.9 mM KH_2_PO_4_, and 5.5 mM glucose), gassed with 95% O_2_–5% CO_2_ to maintain pH to 7.35–7.45 ^38^. Hearts were perfused in a constant perfusion pressure (75 mmHg) mode and paced at 420 beats per minute during the periods of equilibration and reperfusion after 10 min to maintain a constant heart rate. The pacing was stopped during ischemia. Cardiac function was monitored with a balloon inserted into the left ventricle, and data were recorded digitally with Powerlab (AD Instruments, Colorado Springs, CO). After 15 min. of equilibration perfusion, hearts (WT = 8, deletion = 7) underwent 25 min. global ischemia at 37 °C and 30 min. reperfusion (Fig. 2a). Coronary effluent was collected into a beaker placed on ice during the entire 30 min reperfusion. The volume of coronary effluent was measured, and two aliquots were saved for LDH measurement. Hearts (WT = 7, deletion = 7) in the time control groups were only buffer perfused without ischemia. The activity of LDH was measured in collected samples and final LDH activity was normalized with the volume of coronary effluent and heart weight^38^.

Infarct size was measured in another set of mice following ischemia–reperfusion. After 15 min. of equilibration perfusion, hearts (WT = 6, deletion = 8) underwent 25 min. global ischemia at 37 °C and 60 min reperfusion (Fig. 3a). Then, mouse hearts were collected and frozen at − 20 °C for 20 min. Heart tissue was sectioned into 2 mm thick slices and incubated in 1% 2,3,5,-triphenyltetrazolium chloride for 20 min at 37 °C. The slices of heart tissue were stored in 10% formalin overnight. Infarct size was measured using a computerized morphometric system and National Institutes of Health Image software (Image J) to measure areas of infarction and the total area of myocardium. Infarct size was expressed as percentage of the entire myocardium^38^.

### Isolation of mouse heart mitochondria

The mouse heart was removed from the perfusion cannula at the end of perfusion and placed into buffer A [100 mM KCl, 50 mM 3-(*N*-morpholino) propanesulfonic acid (MOPS), 1 mM EGTA, 5 mM MgSO_4_ 7 H2O, and 1 mM ATP, pH 7.4] at 4 °C. SSM (subsarcolemmal mitochondria) and IFM (interfibrillar mitochondria) were isolated from a single mouse heart^75^. Since SSM are more prone to damage during ischemia–reperfusion, potentially due to decreased calcium tolerance^79^, we studied if deletion of CPNS1 can protect this vulnerable population of mitochondria^80^. IFM were isolated for proteomic study in that potential contamination from cytosol was removed during IFM preparation^81^. Cardiac tissue was finely minced and placed in buffer A containing 0.2% bovine serum albumin and homogenized with a polytron tissue processor (Brinkman Instruments, Westbury, NY) for 2.5 s with speed at 10,000 rpm. The polytron homogenate was centrifuged at 500×*g* to generate supernatant and pellet components. The supernatant was centrifuged at 3000×*g* to sediment SSM. The pellet was homogenized again with a polytron tissue processor and incubated with 5 mg/g (wet weight) trypsin for 10 min at 4 °C to release IFM from myofilaments. The homogenate was centrifuged at 500×*g* to generate supernatant that was centrifuged at 3000×*g* to sediment IFM. SSM and IFM were washed twice and then suspended in 100 mM KCl, 50 mM MOPS, and 0.5 mM EGTA. Mitochondrial protein content was measured by the Lowry method, using bovine serum album as a standard^3^.

### Mitochondrial oxidative phosphorylation

The rate of oxygen consumption in mitochondria was measured using a Clark-type oxygen electrode at 30 °C as previously described^82^. Mitochondria were incubated in 80 mM KCl, 50 mM MOPS, 1 mM EGTA, 5 mM KH_2_PO_4_, and 1 mg defatted, dialyzed bovine serum albumin/ml at pH 7.4. Glutamate (20 mM) + malate (10 mM), succinate (20 mM), and TMPD (*N*,*N*,*N*′,*N*′ tetramethyl p-phenylenediamine, 1 mM)-ascorbate (10 mM) were used as complex I, II, and IV substrates, respectively. Rotenone (7.5 μM) was used to block potential reverse electron flow when succinate and TMPD-ascorbate were used as substrates.

### Determination of calcium retention capacity (CRC) in isolated mitochondria

CRC was used to assess the sensitivity of MPTP opening in isolated mitochondria^83^. Mitochondria (125 μg/ml) were incubated in medium containing 150 mM sucrose, 50 mM KCl, 2 mM KH_2_PO_4_, 5 mM succinate in 20 mM Tris/HCl, pH 7.4 by sequential pulses of a known amount of calcium (5 nmol). Succinate was used to energize mitochondria during CRC measurement in that mitochondria can tolerate a greater calcium exposure with succinate as substrate compared to complex I substrate^84^. Extra-mitochondrial Ca^2+^ concentration was recorded with 0.5 µM Calcium Green-5N and fluorescence monitored with excitation and emission wavelengths set at 500 and 530 nm, respectively. Fluorescence spectrometer (LS 55, PerkinElmer, Inc. Waltham, MA) was used for the CRC and following H_2_O_2_ measurement.

### *The production of H*_*2*_*O*_*2*_* in isolated mitochondria*

The net release of H_2_O_2_ from intact mitochondria was measured using the oxidation of the fluorogenic indicator amplex red in the presence of horseradish peroxidase (HRP)^65^. Glutamate (20 mM) + Malate (5 mM) or succinate (20 mM) + Rotenone (5 µM, rotenone was used to block reverse electron flow from complex II to complex I) were used as complex I or complex II substrates, respectively^65^. Maximal H_2_O_2_ generation was determined in the solubilized mitochondria (treated by 5% cholate) with NADH (1 μM) as substrate^65^.

### Proteomic analysis

The purified IFM were used for proteomic study in that potential cytosolic contamination was removed during trypsin treatment^2,22^. Each mitochondrial sample was fractionated and separated on an SDS-PAGE gel, and the identified band was used for in-gel digestion^75^. The peptides were extracted from the polyacrylamide in two aliquots of 30 μl 50% acetonitrile with 5% formic acid. These extracts were combined and evaporated to < 10 μl in Speedvac and then re-suspended in 1% acetic acid to make up a final volume of ~ 30 μl for LC–MS analysis. Digested peptides were analyzed on a ThermoFisher Scientific UltiMate 3000 UHPLC system (ThermoFisher Scientific, Bremen, Germany) interfaced with a ThermoFisher Scientific Orbitrap Fusion Lumos Tribrid mass spectrometer (Thermo Scientific, Bremen, Germany). Liquid chromatography was performed prior to MS/MS analysis for peptide separation^75^. The HPLC column used^14^ is a Thermo Scientific™ Acclaim™ PepMap™ 100 C18 reversed-phase capillary chromatography column (Thermo Fisher Scientific, Waltham, MA) 75 µm × 15 cm, 2 μm, 100 Å^14^. Five μl peptide sample was injected and peptides eluted from the column by a 100-min acetonitrile/0.1% formic acid gradient at a flow rate of 0.30 μl/min and introduced to the source of the mass spectrometer on-line. Nano electrospray ion source was operated at 2.3 kV. The digest was analyzed using the data dependent multitask capability of the instrument acquiring full scan mass spectra using a Fourier Transform (FT) orbitrap analyzer to determine peptide molecular weights and collision induced dissociation (CID) MS/MS product ion spectra with an ion-trap analyzer at 35% normalized collision energy (NCE) to determine the amino acid sequence in successive instrument scans. The MS method used in this study was a data-dependent acquisition (DDA) with 3 s duty cycle. It includes one full scan at a resolution of 120,000 followed by as many MS/MS scans in the ion-trap (35% normalized collision energy)^14^ as possible on the most abundant ions with a 3 s duty cycle. Dynamic exclusion was enabled with a repeat count of 1 and ions within 10 ppm of the fragmented mass were excluded for 60 s.

The data were analyzed using Thermo Scientific Proteome Discoverer (PD) V2.3 (Thermo Fisher Scientific Inc, Waltham, MA, USA) with the search engine Sequest which is integrated in the PD software. The database used to search the MS/MS spectra was the Uniprot mouse protein database containing 16,996 entries with an automatically generated decoy database (reversed sequences). The search was performed looking for fully tryptic peptides with a maximum of two missed cleavages. Oxidation of methionine and acetylation of protein N-terminus were set as dynamic modifications and carbamidomethylation of cysteine was set as a static modification. The precursor mass tolerance for these searches was set to 10 ppm and the fragment ion mass tolerance was set to 0.6 Da. A false discovery rate (FDR) was set to 1% for both peptides and proteins, and two peptides were required for a positive protein identification. Protein quantities were estimated by Minora Feature Detector node in Proteome Discoverer V2.3 using full scan intensities of the identified peptides. These are based on the (raw) intensities that are normalized on multiple levels to ensure that profiles of normalized intensities across samples accurately reflect the relative amounts of the proteins. Missing quantitative values were imputed using low abundance resampling method replacing the missing values with random values sampled between the minimum and the lower 5 percent of all detected values.

The mass spectrometry proteomics data have been deposited to the ProteomeXchange Consortium via the PRIDE^85^ partner repository with the dataset identifier PXD022689 (https://doi.org/10.6019/PXD022689).

### Immunoblotting

Mitochondrial proteins were separated using pre-made 12% or 4–15% Tris–glycine gels (Bio-Rad, Hercules, CA) and transferred to PVDF membrane (Millipore) using semi-dry transfer (Bio-Rad). The blots were incubated for 1 h at room temperature in 5% (w/v) non-fat dry milk (Bio-Rad) in TBST buffer (10 mM Tris pH 7.5, 150 mM NaCl, 0.1% Tween20) followed by the overnight incubation at 4 °C with primary antibody. Primary antibody information is provided in Table 5. After one-hour incubation at room temperature with a 1:5000 dilution of HRP-conjugated anti-mouse or anti-rabbit IgG F(ab)_2_ (GE Healthcare Life Sciences, Piscataway, NJ), blots were developed. Background intensity adjustment, if performed, was always adjusted for the entire membrane. Membranes were cut horizontally in the prepared figures.

**Table 5:** Antibodies used in the current study.

Antibody name	Company	Catalog number	Concentration
AIF	Cell Signaling	4642	1:1000
Cytochrome *c*	ThermoFisher Scientific	710627	1:2500
GAPDH	Cell Signaling	5174	1:1000
PARP	Cell Signaling	9532	1:1000
Spectrin	Santa Cruz	csc-46696	1:100
VDAC	Abcam	14715	1:2500

### Statistical analysis

Data are expressed as the mean ± standard deviation (SD). For all analyses, differences between groups (≥ 3 groups) were compared by one-way ANOVA. When a significant F value was obtained, means were compared using the Student–Newman–Keuls test of multiple comparisons. Differences between two groups were compared by two tail unpaired Student t-test (Version 3.5, Systat Software, Inc., San Jose, CA). Statistical significance was defined as a value of *p* < 0.05.

## Supplementary Information

Supplementary Information.
