# Supplementary figures and images for: Calpain-mediated protein targets in cardiac mitochondria following ischemia–reperfusion
Source: Sci Rep. 2022 Jan 7;12:138. doi: 10.1038/s41598-021-03947-9 (PMC8741987; doi:10.1038/s41598-021-03947-9)

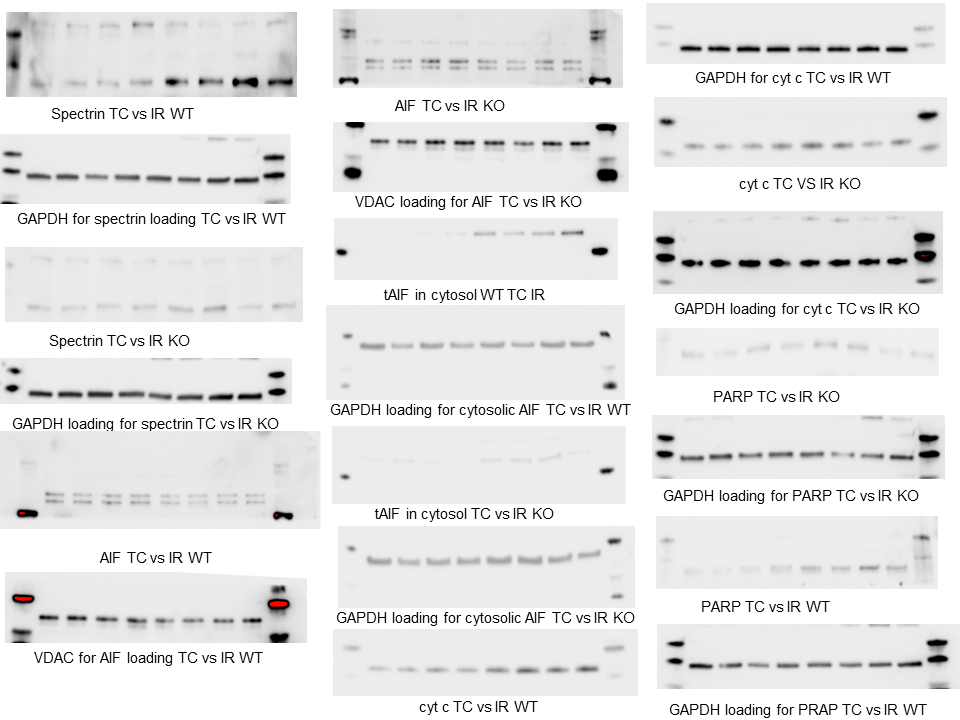

Supplement: Supplementary file 1 — Supplementary Information. [file 41598_2021_3947_MOESM1_ESM.tif]
